# Parametric modeling of deformable linear objects for robotic outfitting and maintenance of space systems

**DOI:** 10.3389/frobt.2025.1565837

**Published:** 2025-07-29

**Authors:** Amy Quartaro, Joshua Moser, John Cooper, Erik Komendera

**Affiliations:** ^1^ FASER Lab, Virginia Tech, Mechanical Engineering Department, Blacksburg, VA, United States; ^2^ Autonomous Integrated Systems Research Branch, NASA Langley Research Center, Hampton, VA, United States

**Keywords:** deformable linear objects, robotic outfitting, model based state estimation, in-space construction, lunar outfitting, parameter estimation

## Abstract

Outfitting and maintenance are important to an in-space architecture consisting of long duration missions. During such missions, crew is not continuously present; robotic agents become essential to the construction, maintenance, and servicing of complicated space assets, requiring some degree of autonomy to plan and execute tasks. There has been significant research into manipulation planning for rigid elements for in-space assembly and servicing, but flexible electrical cables, which fall under the domain of Deformable Linear Objects (DLOs), have not received such attention despite being critical components of powered space systems. Cables often have a non-zero bend equilibrium configuration, which the majority of DLO research does not consider. This article implements a model-based optimization approach to estimate cable configuration, where a design parameter of the model’s discretization level enables trading model accuracy vs computational complexity. Observed 2D cable configurations are used to improve the model via parameter estimation. The parameter estimation is validated through comparing predicted configurations based on estimated parameters to that of a real cable. The incorporation of parameter estimation to the cable model is shown to reduce prediction errors by an order of magnitude. The results of this work demonstrate some of the challenges present with robotic cable manipulation, exploring the complexities of outfitting and maintenance operations of in-space facilities, and puts forth a method for reducing the size of the state space of a cable payload while accounting for non-zero equilibrium configurations.

## 1 Introduction

With the introduction of permanent in-space assets that will not have continuous crew, such as a lunar space station or a surface facility, autonomous robotic systems are critical to infrastructure development and maintenance. This article investigates the modeling of electrical cables, required for robotic construction and maintenance, focusing on minimizing the cable state space and estimating hardware parameters. This is a challenging and unsolved area in the space robotics domain. Cables, which fall under the mechanical domain of Deformable Linear Objects (DLOs), are present in a vast majority of subsystems responsible for providing power and data transfer. This article presents a parametric multibody kinematic formulation for DLOs which balances computational complexity and model accuracy. A parameter estimation scheme leveraging visual measurements is used to increase model accuracy for a given cable. Utilizing image processing techniques, measurements of a cable center-line are obtained to enable parameter estimation and validation of predicted configurations.

Over the last few decades there has been significant research in the field of in-space robotics, spanning a wide variety of projects: demonstrated robotic servicing of spacecraft in low Earth orbit (LEO) (Gao and Chien, 2017; Li et al., 2019), assembly of large aperture telescopes (Belvin et al., 2016; Cresta et al., 2024), and surface robotics for the Moon and Mars (Govindaraj et al., 2019; Zhang et al., 2024; Waltz et al., 2022; Puig-Navarro et al., 2024). Autonomous systems are becoming increasingly important to space and planetary missions where communication latency is high. There have been substantial efforts in autonomous robotic in-space construction and servicing, including multi-robotic operations (Karumanchi et al., 2018; Everson et al., 2020), precision control (Nakanose and Nakamura-Messenger, 2023; Chang and Marquez, 2018), and *in situ* manufacturing (ICON, 2022; Muthumanickam et al., 2022). However, all of these efforts focus on the manipulation of rigid objects or the erection of large scale structures. Long-term space missions will require robotic agents for outfitting and maintenance including manipulation of electrical cables for power and data. Cables require a different approach than rigid elements due to their nature as DLOs. While outfitting is a recognized technology gap by the space community (NASA, 2024), existing work on cable manipulation and outfitting tasks with space applications in mind has been preliminary (Quartaro et al., 2023; Merila et al., 2023; Rojas, 2022).

Robotic DLO manipulation itself is an active research area, with recent findings applied to areas including industrial assembly (Jiang et al., 2010; Hermansson et al., 2013; Shah et al., 2018; Suzuki et al., 2022), surgical applications (Leibrandt et al., 2023; Hu et al., 2024), and soft robotics (Olson et al., 2020; Hammond et al., 2023; Xun et al., 2024; Tummers et al., 2023). For in-space outfitting and maintenance robotic operations, cable routing and installation is often in obstacle-dense, complex environments. Trajectory planners for robotic cable outfitting operations must be able to properly predict the path of the cable as is moves through a space to prevent kinks and snags, violation of bend constraints, and collisions with hazardous objects in the environment. DLOs are considered flexible elements, consisting of infinite degrees of freedom (DOF). This flexibility makes traditional rigid body mechanics difficult to realize without a very large number of states to track in the system, or sacrificing accuracy to simplify the representation. A DLO model formulation cognizant of the effects of large state vectors and model accuracy is needed to provide an approach for robotic DLO manipulation without full teleoperation.

DLOs can be modeled using a variety of methods (Lv et al., 2020). For simple discretized DLO models, the mass-spring approach is common, where point masses act as particle nodes connected by combinations of rotational and torsional springs and dampers (Lv et al., 2017; Yu et al., 2023; Iben et al., 2013). In mutibody spring formulations, the DLO is represented as connected rigid bodies with internal springs and dampers at the joints, generating force based on DLO motion and deflection (Suzuki et al., 2022; Choe et al., 2005). The static DLO configuration is then solved for by minimizing the potential energy in the system (Wakamatsu et al., 1995). More complex approaches to modeling DLOs include Cosserat rod theory (Tummers et al., 2023; Gazzola et al., 2018), position based dynamics (Deul et al., 2018; Liu et al., 2023), and high fidelity Finite Element Methods (FEM) (Lan and Shabana, 2009; Koessler et al., 2021). In recent years, machine learning approaches for DLO models have been explored (Yan et al., 2020; Jin et al., 2022; Zhaole et al., 2024). Approaches such as Cosserat Rod theory and FEM provide accurate results but have high dimensionality and are computationally intractable for real-time systems. Learned models based on training from pre-flight data can be significantly faster than model-based methods; however, a reliance on training data may not correctly account for equilibrium positions once in an extreme environment, adding time and risk to DLO manipulation tasks. The approach taken in this work utilizes a multibody model due to its versatility and the ability to control the trade off between accuracy and the size of the state space. This formulation has been shown to have similar performance to a the more complex Cosserat Model for the DLO configurations considered in this article (Quartaro et al., 2024).

The DLOs in this article are evaluated in the context of outfitting and maintenance, focused on electrical cables. Electrical DLOs are usually composed of multiple wires, insulation, and shielding yielding non-homogeneous cross-sections of metal (primarily copper), plastics, or rubber. Due to factors such as the complexities in manufacturing and containing materials that may undergo plastic deformation, the complexity of cables leads to an accumulation of internal stresses which can cause the DLO to have static equilibrium in a non-zero (bent/curved) configuration; a configuration that must be considered in creating a proper model of a cable. The majority of DLO research focuses assumes an unbent equilibrium position (Lv et al., 2020). Existing efforts to estimate parameters of DLOs either do not address the non-zero equilibrium case (Ying and Yamazaki, 2024; Caporali et al., 2024) or assume the equilibrium points as inputs (Lv et al., 2022; Monguzzi et al., 2025). There has not yet been research conducted into experimentally determining the equilibrium configuration of a DLO, which must be done with both ends constrained for complex environments such as in-space operations.

The multibody model formulated in this article represents cables as inextensible DLOs, built parametrically to provide control over the trade-off between accuracy and complexity via the discretization level of the DLO as a design variable. This article evaluates the case of DLO moving through space with known end points in space, similar to a simple robotic routing case of a single manipulator pulling along a cable. To demonstrate initial model development, both estimation and data collection in this article are done in a 2D plane. The model estimates the DLO configuration by minimizing the potential energy across the DLO length. The potential energy model relies on model parameters representing the physical DLO’s equilibrium positions and stiffness to provide a proper configuration estimate.

Model parameters are estimated through an augmented optimization problem that incorporates point cloud DLO observations. Over multiple configurations, parameter estimation is achieved via a least-squares regression comparing the results of the optimization to the configuration required to satisfy the Lagrangian mechanics. The configuration and parameter estimates are dependent on each other, and are found iteratively until the estimate of both converges. By estimating DLO parameters it is then possible to predict behavior of a DLO with a non-zero equilibrium configuration without measurements, for applications such as trajectory planning or movement through occluded regions. Prediction results highlighted in this article demonstrate the viability of the proposed approach and advance the state of the art of DLO modeling for robotic in-space outfitting and maintenance operations.

This article is organized as follows: Section 2 describes the parametric model for estimating the cable position based on predicted potential energy, as well as the estimation of physical parameters to increase accuracy of the specific cable asset to be modeled. Section 3 describes a hardware validation setup, the procedure to measure a static cable point-cloud, and compares performance of different discretization levels in the model. Section 3 also explores the validation of the proposed model through configuration prediction. Finally, a discussion on further work and applications to space systems is discussed in Section 4.

## 2 Deformable linear object model

This section details the formulation of the kinematics, energy optimization, and parameter estimation techniques used to model the cable as a DLO. The DLO is represented as a series of rigid links connected at node end points. The nodes contain torsional springs and dampers which generate internal forces. This work is focused on the static case, and therefore dampers are not considered in the remainder of the formulation. The number of links in the DLO, n , is a design parameter that dictates the size of the model state space and can be tuned to balance estimation accuracy and number of calculations required. System parameters consist of the DLO stiffness and equilibrium positions, which are determined through observations and are dependent on n .

**FIGURE 1:**
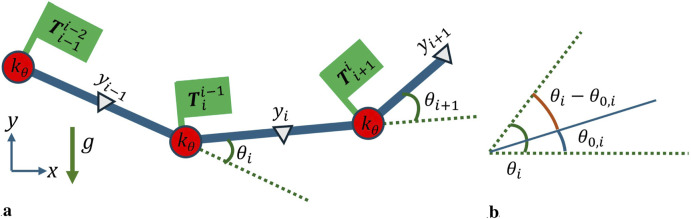
Multibody system definition **(a)** Geometry of the links, where the circles indicate the nodes and their corresponding springs with stiffness $k_{\theta }$ , $\theta_{i}$ indicates relative rotation, the triangles indicate the $y_{i}$ position used for gravitational potential energy, and the link transforms $T_{i}^{i-1}$ are marked to indicate link position and rotation from frame i−1 to from i . **(b)** For a link rotation angle $\theta_{i}$ , there is a reference angle $\theta_{0,i}$ such that the rotational deformation in the link is $\theta_{i}-\theta_{0,i}$ **(a)** DLO link definition. **(b)** Offset angle for strain energy calculation.

The DLO is split into n links, each connected by a start and end node, represented as circles in Figure 1a. The start node of a link is used as the location of the links frame for the kinematic formulation. Section 2.1 defines the kinematics of the DLO based on relative joint angles, with the constraint that the end node of the $i^{th}$ node is coincident with the start node of the ${\left(i+1\right)}^{th}$ node. The DLO is assumed to have constant diameter, uniform mass, and a known length. The link mass, $m_{\mathrm{link}}$ , is constant and defined as a fraction of the total cable mass, $m_{\mathrm{link}}=\frac{m_{\mathrm{cable}}}{n}$ . Similarly, the link length, $l_{\mathrm{link}}$ , is dependent on the total length and the number of links in the cable, $l_{\mathrm{link}}=\frac{l_{\mathrm{cable}}}{n}$ . The rotational stiffness constant of the springs at each node, $k_{\theta }$ , is considered equal for all springs and is approximated based on the DLO geometry and material. Gravitational forces act in the −y direction; the height of the $i^{th}$ link center of mass (COM), $y_{i}$ , is used to calculate the potential energy due to gravity in the cable, indicated with triangles in Figure 1a.

Section 2.2 defines how these properties of the DLO can be leveraged to understand the potential energy stored in the system. Section 2.2.1 formulates the optimization problem used to estimate the DLO configuration using joint angles by minimizing the potential energy in the system subject to kinematic constraints.

Section 2.3 augments the objective function of the optimization problem to incorporate point cloud measurements, enabling parameter estimation. Parameter estimation can be achieved using this augmented optimization problem and measurements of the DLO in multiple configurations. Parameters are adjusted iteratively to reduce distance error and ensure valid configurations across the dataset, as presented in Section 2.4. The parameter estimation scheme enables the model to predict DLO configurations for a non-zero equilibrium positions, benefiting efforts to plan for robotic manipulation of DLOs in high risk environments, including spaceflight systems.

A summary of the notation used throughout this section to formulate the pose and parameter estimation algorithms can be found in Table 1. Variables that are presented in bold are vectors or matrices, with lowercase indicating vectors ( p ) and uppercase indicating matrices ( $T_{j}^{i}$ ). Otherwise the variable is a scalar . Standard SI units are used in this formulation.

**TABLE 1:** DLO model notation reference.

Variable	Definition	Set
$\hat{\left[\cdot \right]}$	Variable in $\hat{\left[\cdot \right]}$ represents an estimated quantity	-
$\tilde{\left[\cdot \right]}$	Variable in $\tilde{\left[\cdot \right]}$ represents a measured quantity	-
n	Number of links in cable, discretization	Z
m	Number of configurations for parameter estimation	Z
a,i,j,k	Iteration counters	Z
$m_{\mathrm{cable}}$	Total cable mass [kg]	R
$m_{\mathrm{link}}$	Mass per link [kg]	R
$l_{\mathrm{cable}}$	Total cable length [m]	R
$l_{\mathrm{link}}$	Length per link [m]	R
$k_{\theta }$	Approximate bending stiffness of rotational joints [Nm/rad]	R
g	Gravity magnitude, assumed g=9.81 in the -y direction [m/s^2^]	R
β	Weighting factor for distance residual [Nm/m]	R
$\theta_{i}$	Relative angle between link i−1 and i [rad]	R
$\theta_{0,i}$	Zero-strain reference angle between link i−1 and i [rad]	R
V(θ)	Potential energy function [J or Nm]	R
θ	Generalized coordinate vector containing all relative angles θ [rad]	$R^{n+1}$
${\theta^{\prime }}_{i}$	Reduced coordinate vector containing relative angles up to $\theta_{i}$ [rad]	$R^{i}$
$\theta_{0}$	Zero-strain reference vector containing all reference angles $\theta_{0,i}$ [rad]	$R^{n+1}$
α	Parameter vector for cable	$R^{n+2}$
q	Generalized coordinate vector of all θ values across m configurations [rad]	$R^{\left(n+1\right)m}$
$p_{j}^{i}$	Cartesian position vector from point i to frame j [m]	$R^{3}$
$R_{j}^{i}$	SO(3) rotation matrix from frame i to frame j	$R^{3\times 3}$
$T_{j}^{i}$	SE(3) transformation matrix from frame i to frame j	$R^{4\times 4}$
$e_{i}$	Standard basis vector with $i^{th}$ index equal to one	$R^{4}$

### 2.1 Multibody DLO kinematics

The rotations between links are the independent variables in this problem that define the kinematics of the system. For the planar case considered in this article, links are defined as revolute joints with 1 rotational DOF per joint. These rotations are represented as a single generalized coordinate vector θ , shown in Equation 1.

$$\theta =\left[\begin{array}{ccc} \theta_{1} & \ldots \theta_{i}\ldots & \theta_{n+1} \end{array}\right]\in R^{n+1}\;\text{for }i=1\ldots n+1$$ (1)

The variable $\theta_{i}$ represents the relative rotation between the ${\left(i-1\right)}^{th}$ and $i^{th}$ links. The DLO is assumed to be inextensible. The $\theta_{n+1}$ value represents a spring that connects the DLO to the desired end point, at the end node of link n . The multibody kinematic formulation is shown in Figure 1.

This formulation utilizes homogeneous transformation matrices T∈SE(3) to represent the spatial position and orientation of DLO links. The SE(3) representation of position and orientation is used to represent DLO configuration. The SE(3) transformation matrix $T_{j}^{i}\in R^{\mathrm{4x4}}$ , defined in Equation 2, contains the position and orientation of a link from frame *i* to frame *j*:

$$T_{j}^{i}=\left[\begin{array}{cc} R_{j}^{i} & p_{j}^{i}\\ 0 & 1 \end{array}\right]$$ (2)

Where $R_{j}^{i}\in SO\left(3\right)$ and $p_{j}^{i}\in R^{3}$ are the rotation matrix and Cartesian position vector from frame i to frame j , respectively. The global frame containing the test origin is defined as frame 0.

The boundary conditions in this article assume that the DLO is *pinned* to the prescribed start and end points. The pinned constraint indicates that the location is fixed but the rotation is free and does not contribute potential energy to the system. The pinned constraint might be achieved by a DLO containing grappling fixtures for a robot to interface with as a way to reduce stress in the DLO. The pinned boundary condition also benefits this work by reducing the impact of errors in the test setup used to evaluate model performance. The start node aligns with the global frame, $p_{1}^{0}={\left[\begin{array}{ccc} 0 & 0 & 0 \end{array}\right]}^{T}$ , and the DLO end point must align with the desired position, $p_{n+1}^{0}\left(\theta \right)=p_{\mathrm{des}}$ .

$T_{i}^{0}$ is the configuration of the base node of link i with respect to the global frame. As the $i^{th}$ node location is dependent on the current and preceding θ coordinates, a reduced coordinate vector is used for the calculation of $T_{i}^{0}$ , $\theta_{i}^{\prime }$ , defined in Equation 3.

$$\theta_{i}^{\prime }=\left[\begin{array}{ccc} \theta_{1} & \ldots & \theta_{i} \end{array}\right]\in R^{i}$$ (3)

Therefore, the configuration of a given node is as defined in Equation 4. The location of the base node and $T_{i}^{0}$ for a given link is shown in Figure 1a.

$$\begin{array}{ccccc} T_{i}^{0}\left({\theta^{\prime }}_{i}\right) & =T_{1}^{0}\left(\theta_{1}\right)\overset{i}{\underset{a=2}{\prod }}T_{a}^{a-1}\left(\theta_{a}\right)\\ \text{where}\\ T_{1}^{0}\left(\theta_{1}\right) & =\left[\begin{array}{cc} R_{1}\left(\theta_{1}\right) & 0\\ 0 & 1 \end{array}\right], & & T_{a}^{a-1}\left(\theta_{a}\right) & =\left[\begin{array}{cc} R_{a}^{a-1}\left(\theta_{a}\right) & l_{\mathrm{link}}\\ 0 & 1 \end{array}\right] \end{array}$$ (4)

The position vector $l_{\mathrm{link}}={\left[\begin{array}{ccc} l_{\mathrm{link}} & 0 & 0 \end{array}\right]}^{T}$ is a constant vector describing the geometry of the link, and is the same for every link in the cable. The first link must be coincident with the global origin at its start node, instead of a preceding link, resulting in a rotation but no translation of frame 1. This first link differs from all remaining links, reflected in Equation 4.

The global location of each link’s COM is needed to understand the potential energy due to gravity forces. In this formulation gravity is assumed to act in the −y direction, therefore only the y -coordinate of link i ’s COM in the global frame, $y_{i}$ , contributes to the gravitational energy. $y_{i}$ is a function of the relative link rotations ${\theta^{\prime }}_{i}$ and is found through Equation 5.

$$y_{i}\left({\theta^{\prime }}_{i}\right)=T_{i}^{0}\left({\theta^{\prime }}_{i}\right)\left[\begin{array}{c} 0.5l_{\mathrm{link}}\\ 1 \end{array}\right]e_{2}$$ (5)

where $e_{j}\in R^{4}$ is a standard basis vector. Each link is considered to have uniform mass, leading to the center of mass being at half the length of the link, $0.5l_{\mathrm{link}}$ .

### 2.2 Potential energy formulation

At rest, a DLO is at a minimal potential energy state. The total potential energy in the DLO, V(θ) , is as defined in Equation 6.

$$V\left(\theta \right)=\overset{n}{\underset{i=2}{\sum }}\left[\frac{1}{2}k_{\theta }{\left(\theta_{i}-\theta_{0,i}\right)}^{2}+m_{\mathrm{link}}gy_{i}\left({\theta^{\prime }}_{i}\right)\right]+m_{\mathrm{link}}gy_{1}\left(\theta_{1}\right)$$ (6)

The first and second terms of the summation in Equation 6 are contributions from the spring strain energy and gravitational potential energy, respectively. There are n+1 torsional springs for n link segments, $k_{\theta }$ is the DLO stiffness, $\theta_{i}$ is the rotation angle from the i−1 to i link segment and $\theta_{0,i}$ is the zero-strain relative rotation of the node. The relationship between $\theta_{i}$ and $\theta_{0,i}$ is emphasized in Figure 1b. A non-zero $\theta_{0,i}$ is indicative of a bent equilibrium configuration. $m_{\mathrm{link}}$ is the mass of an individual link, g is the magnitude of the gravity force (assumed in the -y direction) and $y_{i}\left(\theta \right)$ is the y-position of the $i^{th}$ link COM, defined in Equation 5. The starting node of the first link does not contribute strain energy, but link still has length and mass, hence the addition of the $y_{1}$ gravitational potential term after the summation. The n+1 spring does not have a link mass associated with it, and does not contribute strain energy due to the pinned boundary condition; the n+1 spring is therefore not included in Equation 6.

In the DLO formulation used in this work, the forces contributing to the DLO’s energy are all conservative forces. Lagrange’s equation states that if a system is at rest (no kinetic energy), the gradient of the potential energy with respect to the generalized coordinates, $\nabla_{\theta }V\left(\theta \right)$ , must be equal to zero (?). The gradient for the DLO’s potential energy is shown in Equation 7.

$$\begin{array}{cc} \nabla_{\theta }V\left(\theta \right) & =0=k_{\theta }\left(\theta -\theta_{0}\right)+\overset{n}{\underset{i=1}{\sum }}m_{\mathrm{link}}g\nabla_{\theta }y_{i}\left(\theta \right) \end{array}$$ (7)

where $\nabla_{\theta }y_{j}\left(\theta \right)$ describes the gradient of the DLO with respect to the generalized coordinates θ .

#### 2.2.1 Potential energy minimization

The DLO generalized coordinates θ can be found by minimizing the total potential energy in the system, as in Equation 6. The minimization is subject to constraints enforcing the start and end point boundary conditions, where the start point must be coincident with the global frame origin and the end point, $p_{n+1}^{0}\left(\theta \right)$ , must be coincident with the desired end point, $p_{\mathrm{des}}$ . $p_{n+1}^{0}\left(\theta \right)$ is the position vector of $T_{n+1}^{0}\left(\theta \right)$ defined by Equation 4. Equation 8 illustrates the potential energy optimization problem.

$$\begin{array}{cc} \min_{\theta } & \;f_{0}\left(\theta \right)=V\left(\theta \right)=\overset{n}{\underset{i=0}{\sum }}\frac{1}{2}k_{\theta }{\left(\theta_{i}-\theta_{0,i}\right)}^{2}+mgy_{i}\left(\theta \right)\\ \text{subject to } & \;f_{1}\left(\theta \right)=p_{n+1}^{0}\left(\theta \right)-p_{\mathrm{des}}=0\\ & \;\;-\frac{\pi }{2}⪯\theta ⪯\frac{\pi }{2} \end{array}$$ (8)

The generalized coordinates are constrained as an assumption of how the DLO behaves, maximum bend requirements could easily be added to Equation 8. To solve the problem posed in Equation 8 this article leverages an off-the-shelf numerical solver method, *COBYLA*, as implemented in (Virtanen et al., 2020). In this formulation the inclusion of gravity results in one solution for the minimization, if gravity is considered negligible (a possible case for in-space operations), additional constraints are necessary to ensure there is one solution for the potential energy minimization problem.

The configuration generated through solving Equation 8 is the expected position based on given end points and cable parameters. These inputs allow for the model to predict DLO behavior as the end positions change, i.e., the DLO is moved through the workspace. However, the accuracy of the configuration estimate is reliant on the accuracy of the model parameters for stiffness and equilibrium configuration. If the parameters are incorrect, the modeled DLO will not match a real system.

### 2.3 Measured distance minimization

A configuration estimate informed by observations in the case of incorrect parameters is required in order to evaluate the parameters themselves. The optimization problem in Equation 8 can be augmented to take advantage of DLO measurements in addition to the theoretical minimum potential energy configuration. This measurement-infused objective function ensures model nodes of a DLO configuration are pulled towards the correct positions, allowing for the model parameters to be adjusted to reflect the real DLO. Such an objective function is beneficial to in-space applications where a DLO’s parameters may have been impacted by travel and storage before the operation.

Observational data takes the form of a 2D point cloud representing the centerline of the DLO, $\tilde{p}\in R^{l\times d}$ , where *d* is the dimension of the measurement (2 or 3) and l>n is the number of points in the point cloud in the global frame. The point cloud is determined via an image processing procedure detailed in Section 3.1. The estimated point list, $\hat{p}\left(\theta \right)$ , is a concatenated list of the global position vector component of each link frame, $T_{i}^{0}\left({\theta^{\prime }}_{i}\right)$ , as defined in Equation 4. To minimize the distance error between the configuration defined by the generalized coordinates, $\hat{p}\left(\theta \right)$ , and the observed centerline configuration, $\tilde{p}$ , the square of the distance between these points is used as the model residual. The residual term $r_{p}$ is as shown in Equation 9.

$$r_{p}\left(\theta \right)=\left(\|\hat{p}\left(\theta \right)-{\tilde{p}}^{*}{\|}^{2}\right)$$ (9)

Where $\tilde{p}*\in R^{n\times d}$ is a reduced set of points from $\tilde{p}$ determined as the points of the point cloud with the minimum Euclidean distance to $\hat{p}\left(\theta \right)$ . In some cases, where the DLO can only be partially observed, the residual can still be calculated for less than n points.

To create an objective function including terms for both potential energy and the measurement residual, the objective function $f_{0}$ in the optimization problem in Equation 8 is modified. This new objective function, ${f*}_{\;0}\left(\theta \right)$ , is defined in Equation 10. This new objective function replaces $f_{0}\left(\theta \right)$ in the problem posed in Equation 8 to consider measurements. The variables and constraints remain the same.

$$\begin{array}{cc} f_{0}^{*}\left(\theta \right) & =f_{0}\left(\theta \right)+\beta \left(n\right)r_{p}\left(\theta \right)\\ & =\left(V\left(\theta \right)+\beta \left(n\right)\|\hat{p}\left(\theta \right)-{\tilde{p}}^{*}{\|}^{2}\right) \end{array}$$ (10)

The weighting factor β is introduced to allow for design control of how impactful the incoming measurements are on the static estimate. In this work the best performance occurred when β brought the residual $r_{p}$ term to the same order of magnitude as the potential energy term. β was made a function of the number of links in the DLO, becoming β(n)=cn , where c is an experimentally determined constant factor.

Utilizing ${f*}_{0}$ allows for the DLO model to estimate the configuration where parameters are unknown or incorrect but measurements are available. Only after parameters are known can Equation 8 accurately predict the behavior of the DLO in order to plan a proper trajectory with changing end points.

### 2.4 Parameter estimation

Due to uneven cross sections, environmental conditions, or manufacturing errors, cables differ from rope-like DLOs in that they often contain non-zero joint equilibrium configurations. The same inconsistencies can also impact bending stiffness. An estimate of the joint equilibrium positions and stiffness allows for the model to generate predictions that account for these inconsistencies, reducing uncertainty in the model.

In order to estimate equilibrium and stiffness parameters, multiple configurations of the DLO must be considered. Each configuration consists of the generalized coordinate vector, θ , that contains the joint positions of the cable configuration. The configurations are combined to form the complete dataset coordinate vector q , as shown in Equation 11.

$$q=\left[\begin{array}{ccc} \theta_{1} & \ldots \theta_{j}\ldots & \theta_{m} \end{array}\right]\in R^{\left(n+1\right)m}\;\text{for }j=1\ldots m$$ (11)

Where m is the total number of configurations used for parameter estimation; θ is defined by Equation 1.

The parameter values to be estimated include the cable stiffness, $k_{\theta }$ , and equilibrium positions of each segment, $\theta_{0}$ , in the parameterized cable. These parameters are combined into the parameter vector α as shown in Equation 12. The parameter vector α is constant across all cable configurations.

$$\alpha =\left[\begin{array}{ccccc} k_{\theta } & \theta_{0,1} & \ldots & \theta_{0,n} & \theta_{0,n+1} \end{array}\right]\in R^{n+2}$$ (12)

To estimate the values of α , a nonlinear least squares algorithm is used, treating the vector q as the observation vector. Because the potential energy is known to vary based on the parameters contained in α , Equation 6 can be referred to as V(θ,α) . Results from the optimization problem in Section 2.3 provide expected values of $q=\left[\begin{array}{c} \ldots \theta_{j}\ldots \end{array}\right]$ through minimizing Equation 10 for some initial guess of the parameter vector, $\alpha^{0}$ , shown in Equation 13.

$$\theta_{j}=\min_{\theta }\left(V\left(\theta ,\alpha^{0}\right)+\beta \left(n\right)r_{p}\left(\theta \right)\right)\;\text{for}\;\theta_{j}\in q$$ (13)

All coordinate vectors in q utilize the same α , highlighted by the change in notation in the potential energy function. Only one configuration at a time is generated from Equation 13 to emphasize that the configurations in q do not influence each other. The estimated configuration based on the parameters to compare $\theta_{j}$ against, $\hat{\theta }\left(\alpha \right)$ , is determined by rewriting Equation 7 into Equation 14. Because all points in q are measured from static configurations, Equation 14 must hold.

$${\hat{\theta }}_{j}\left(\alpha \right)=\theta_{0}-\overset{n}{\underset{i=1}{\sum }}\frac{m_{\mathrm{link}}g}{k_{\theta }}\nabla_{\theta_{j}}y_{i}\left(\theta_{j}\right)$$ (14)

The ${\hat{\theta }}_{j}\left(\alpha \right)$ for each configuration in the dataset can be combined into the estimate vector $\hat{q}\left(\alpha \right)=\left[\begin{array}{ccc} \ldots & {\hat{\theta }\left(\alpha \right)}_{j} & \ldots \end{array}\right]$ . The objective of the nonlinear least squares problem can then be formulated into an equation for the squared residual, shown in Equation 15.

$$r_{\alpha }\left(\alpha \right)=\|q-\hat{q}\left(\alpha \right){\|}^{2}$$ (15)

A numerical non-linear least squares solver is leveraged in this work, utilizing the trust region reflective method as formulated in Branch et al. (1999) (Virtanen et al., 2020). The standard least squares loss function was sufficient for estimating parameters in this work. In the case of a more complex model, including an increase in constraints or additional external loading, it may be necessary to implement a quadratic loss term or explore probabilistic estimation approaches (such as maximum likelihood estimation).

#### 2.4.1 Iterative estimation loop

The method used to generate q , Equation 8, requires α , and conversely the solution for α is dependent on the contents of q . Therefore, an iterative process between modeling the cable configuration and parameter estimation is performed until the parameter values no longer change between iterations. Algorithm 1 details the iterative process used to generate a final α vector, $\alpha^{f}$ .

Algorithm 1 Least Squares Parameter Estimation

1: $\theta^{0},\alpha^{0}=\left[k_{\theta }^{0},\theta_{0}^{0}\right]$

2: k←0

3: **while** $|\alpha^{k}-\alpha^{k-1}|⪯\epsilon_{\alpha }$ **do**
4:   **for** $\theta_{j}^{k}\in q^{k}$ **do**
5: $\theta_{j}^{k+1}\leftarrow \min_{\theta }\left(V\left(\theta ,\alpha^{k}\right)+\beta \left(n\right)r_{p}\left(\theta \right)\right)$

6:   **end for**
7: $\alpha^{k+1}\leftarrow \min_{\alpha }\left({\left\|q^{k+1}-\hat{q}\left(\alpha \right)\right\|}^{2}\right)$

8: k←k+1

9:  **end while**
10: **return** $\alpha^{f}\leftarrow \alpha^{k}$

Initial values are required for both position and parameters to begin the loop. The iteration loop continues until the absolute value change in α remains greater than a desired tolerance, $\epsilon_{\alpha }$ . In practice, to ensure that the estimated parameters have reached a finite value, the while loop is run until $\epsilon_{\alpha }$ has been satisfied in multiple iterations. In a given iteration, each configuration $\theta^{k}$ in $q^{k}$ is evaluated independently, as the results of the configuration estimate are based on the potential energy in a single configuration. Line 5 calculates the joint angles as in Section 2.3 for the current iteration.

After $q^{k+1}$ has been populated, the parameters that best fit the data can be estimated. Line 7 uses the configuration $q^{k+1}$ compared against the theoretical estimate from Equation 10, ${\hat{q}}^{k}$ , to generate the next iteration of parameters $\alpha^{k+1}$ by minimizing the result of Equation 15. The parameter vector $\alpha^{f}$ produced from Algorithm 1 can be used in predicting future positions of the DLO article utilizing the optimization problem in Section 2.2.

## 3 Experimental results

A hardware experiment was preformed to validate the DLO model and parameter estimation. An electrical cable was mounted to a vertical frame, with prescribed attachment points allowing for a pinned joint. The pinned joint was accomplished by placing a bolt through the center of the cable, and attaching that bolt to the vertical frame. Spacers were used to ensure the cable did not touch the frame for every configuration. The same cable was used to collect image data in 12 different configurations. After a cable was mounted in a known configuration, images that contained the entire cable in each frame were collected and used to generate point clouds of the cable centerline, through the procedure detailed in Section 3.1, to form the dataset used to evaluate the DLO model and parameter estimation performance. Collected point clouds were used to evaluate the model in three different aspects: 1) Impacts of parameter estimation across dataset, 2) How the parameterization of the number of links impacts accuracy and computation time, and 3) prediction performance of parameter informed model for positions not included in the parameter estimation.

The same initial guesses for the model configuration and parameters are used for every configuration. The initial guess for the orientation, $\theta^{0}$ , of every configuration is $\theta_{1}^{0}=-\frac{\pi }{2}$ and all other values in $\theta^{0}$ equal to 0.001.

The initial guess for stiffness is approximated material bending stiffness as a function of the number of links n , $k_{\theta }^{0}\left(n\right)=EI/l_{\mathrm{cable}}\times n$ , where E is an estimated Young’s modulus of the cable, I is the area moment of inertia of the cable cross-section, $l_{\mathrm{cable}}$ is the total cable length, and n is the number of links. The estimated Young’s modulus is calculated utilizing the primary components in the cable test article, copper wire and rubber insulation, assuming a cross-section of 50% wire and 50% insulation. This stiffness approximation for $k_{\theta }$ is used to relate the multibody spring model to the beam stiffness that would be utilized if the cable was treated as a slender beam. The initial guess for equilibrium reference angles $\theta_{0}^{0}$ is all zeros, which would be the ideal equilibrium state for a DLO.

Basic properties of the test cable are documented in Table 2. Computations were completed using Python 3.10, leveraging the scipy (Virtanen et al., 2020) and OpenCV (Bradski, 2000) libraries for the optimization methods and image processing, respectively.

**TABLE 2:** Test cable properties.

Length	0.812 [m]
Diameter	12.8e-3 [m]
Mass	0.23 [kg]
Approximated $k_{\theta }$	94.956 n [Nm]

### 3.1 Image processing

**FIGURE 2:**
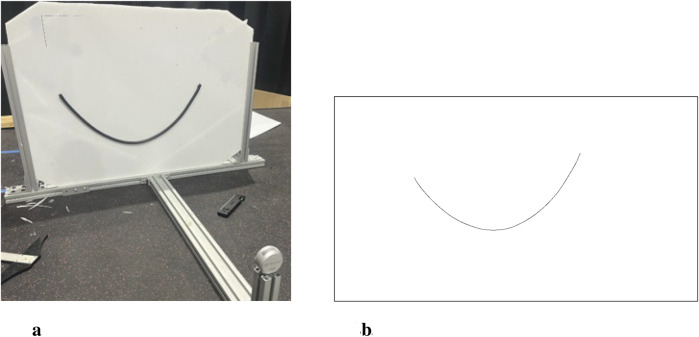
Hardware experiment configuration **(a)** Test stand with mounted cable article **(b)** Processed image containing centerline point cloud used as measurement points $\tilde{p}$ **(a)** Hardware experiment setup. **(b)** Processed measurement image.

For a specific static cable, images of different configurations are captured. These images can then be processed into point clouds of the cable center line to use as measurements in the formulation of the DLO model. There has been significant research into the perception of DLOs with visual observations (Caporali et al., 2022; Tang et al., 2017), however it is not within the scope of this model formulation work to incorporate online DLO perception. As such, the image processing used in this analysis was done offline with respect to the parameter estimation model. True cable shape is obtained using an Intel RealSense D435i camera for RGB images of size 1280x720. The test setup is pictured in Figure 2a. The camera is fixed relative to the vertical stand. The vertical stand consists of fixture points for the DLO and markings to indicate the bounding box used to straighten the images for processing.

The RGB images for a given configuration are processed using the OpenCV library (Bradski, 2000). Edge detection and morphology techniques are used to isolate the cable as a contour in the image. Then, the Zhang-Suen thinning algorithm (Zhang and Suen, 1984) is applied via the OpenCV *ximagproc* module to generate the cable centerline to compare against the simulated cables. Figure 2b illustrates the centerline resulting from the processing to provide the measurement point list $\tilde{p}$ .

The DLO model and parameter estimation are compared against $\tilde{p}$ to estimate: the accuracy of the configuration estimate before and after parameter estimation, effects of varying the discretization parameter n , and accuracy of DLO prediction with a pre-compiled α .

### 3.2 Parameter estimation

By performing parameter estimation based on the processed image data, the accuracy and reliability can be significantly improved. The best results were seen when β(n) was set with c=10000 to make the contributions from the measurements to the objective function the same magnitude as those from the potential energy results. Table 3 illustrates how the parameter estimation process is capable of solving for $\theta_{0}$ equilibrium values and stiffness $k_{\theta }$ in the cable from an initial guess $\alpha^{0}$ . As the first and last nodes are pinned, Table 3 does not include $\theta_{0,1}$ and $\theta_{0,n+1}$ as they do not change. The change in the stiffness is minimal across all discretization levels evaluated, except for the case n=5 in which the stiffness estimate doubles to be equal to the $k_{\theta }$ for n=10 . When the approximated stiffness shown in Table 2 is modified slightly for different material compositions, the parameter estimation consistency reaches the same order of magnitude but often not the same exact value. This inconsistency may be due to the cable stiffness being variable across the cable length, which is not the assumption made in this work. Additional research is needed to further investigate the stiffness, and how sensitive the DLO model is to slight deviations of the same order of magnitude of $k_{\theta }$ .

**TABLE 3:** Change in parameters, n=10 .

	$k_{\theta }\left[Nm\right]$	$\theta_{0}\in R^{n-1}$ [rad]								
Initial $\alpha^{0}$	949	0.0	0.0	0.0	0.0	0.0	0.0	0.0	0.0	0.0
Final $\alpha^{f}$	949	0.10	0.19	0.26	0.31	0.32	0.29	0.23	0.17	0.09

**FIGURE 3:**
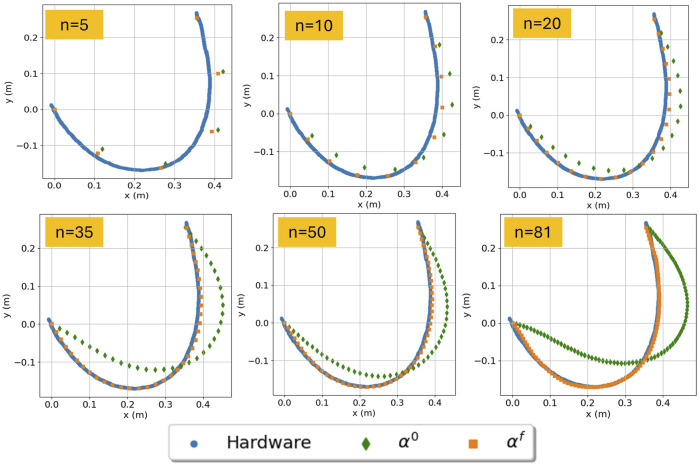
Cable segmentation results of varying the discretization parameter n . Results include both those using initial parameters $\alpha^{0}$ and those at the conclusion of Algorithm 1, using $\alpha^{f}$ . Same end pose, $p_{\mathrm{des}}=\left(0.36,0.25\right)$ [m], for different numbers of segments n=5,10,20,35,50,81 .

The different end points of the dataset, listed in Table 4, have differing errors from the potential energy solutions solved with the initial guess, $\alpha^{0}$ , based on how significant the curvature of the cable is, or when the cable is nearly vertical. Table 4 demonstrates the effects of using either $\alpha^{0}$ or $\alpha^{f}$ as defined in Table 3. The potential energy changes significantly based on the choice of parameters, demonstrated in the V(θ,α) columns of Table 4. The configuration found through Equation 8 with $\alpha^{0}$ produces a minimum energy solution, $V\left(\theta^{0},\alpha^{0}\right)$ , but contains errors when compared to hardware observations (as seen in Figure 3). The second potential energy column contains the potential energy at the final configuration, $\theta^{f}$ , with the estimated parameter vector, α . $V\left(\theta^{f},\alpha^{f}\right)$ , has significantly reduced and error to hardware across the dataset. The third potential energy column contains the values for configuration $\theta^{f}$ , which has lower error to the hardware than $\theta^{0}$ , given the initial parameters instead, $V\left(\theta^{f},\alpha^{0}\right)$ . For every configuration in the dataset, $V\left(\theta^{0},\alpha^{0}\right)<V\left(\theta^{f},\alpha^{0}\right)$ ; without parameter estimation the model in Equation 8 will not converge on the observed positions for this article.

**TABLE 4:** Potential Energy (V), Root Mean Square Error (RMSE) and Standard Deviation (STD) for Evaluated Cable used for Parameter Estimation, before and after implementing adjustment. n=10 .

Configuration	V [Nm]			RMSE [m]		STD [m]	
End Pose [m]	$\left(\theta^{0},\alpha^{0}\right)$	$\left(\theta^{f},\alpha^{f}\right)$	$\left(\theta^{f},\alpha^{0}\right)$	$\alpha^{0}$	$\alpha^{f}$	$\alpha^{0}$	$\alpha^{f}$
(0.36, 0.25)	489.94	55.46	510.09	0.024	0.007	0.011	0.004
(0.51, 0.0)	386.07	19.82	405.74	0.002	0.003	0.001	0.002
(0.51, 0.10)	372.00	16.35	509.23	0.005	0.002	0.003	0.001
(0.51, 0.25)	303.48	6.15	308.63	0.012	0.005	0.006	0.003
(0.51, −0.10)	371.78	17.16	372.71	0.004	0.003	0.002	0.002
(0.61, 0.0)	247.78	0.20	250.10	0.003	0.003	0.002	0.001
(0.61, 0.10)	236.92	−0.10	241.47	0.006	0.003	0.004	0.002
(0.61, −0.10)	236.69	−0.24	266.77	0.003	0.003	0.002	0.002
(0.66, −0.25)	123.58	16.78	124.44	0.004	0.004	0.002	0.002
(0.66, 0.20)	144.51	10.26	146.06	0.005	0.003	0.003	0.002
(0.71, 0.0)	119.41	18.36	191.40	0.004	0.004	0.002	0.002
(0.13, −0.79)	16.22	122.66	20.30	0.121	0.025	0.055	0.012

The remaining columns in Table 4 demonstrate the significant effect calculating parameters has on correcting the configuration estimate, both in the Root Mean Square Error (RMSE) and Standard Deviation (STD) across the nodes in a configuration at the output of Algorithm 1. The parameter estimation significantly drives down the errors across the dataset, with the most significant reductions being an order of magnitude (from cm to mm) in RMSE for multiple configurations, with an average increase in accuracy of 11 mm.

### 3.3 Parameterization

While the formulation and parameter estimation are satisfactory for a wide range of discretization levels, the ability to modify the number of link segments in the model is critical to creating a real-time DLO manipulation architecture. The computation required to generate the estimated angles and parameters from a zero initial guess is significant. While the parameters could be evaluated offline in a real-time environment, computing the configuration for planning purposes should maintain a minimal state vector when possible.

Figure3 illustrates how the estimate changes by changing the discretization parameter. For all cases of n , the configuration estimate that included the distance objective function and estimated $\alpha^{f}$ aligned well with the measured data. In contrast, the configuration estimate using $\alpha^{0}$ degraded in performance as n increased. Table 5 describes the average computation time and accuracy across all configurations, given $\alpha^{f}$ , for varying n .

**TABLE 5:** Root Mean Square Error (RMSE) and Standard Deviation (STD) of parameter estimation results for different choices of n .

n	$l_{\mathrm{link}}$ [m]	Average pos. Est. Time [s]	RMSE [m]	STD [m]
5	0.162	0.2	0.0095	0.0072
10	0.081	0.5	0.0082	0.0068
20	0.041	1.9	0.0078	0.0067
35	0.023	5.9	0.0078	0.0067
50	0.016	7.9	0.0077	0.0067
81	0.010	12.7	0.0081	0.0072

**FIGURE 4:**
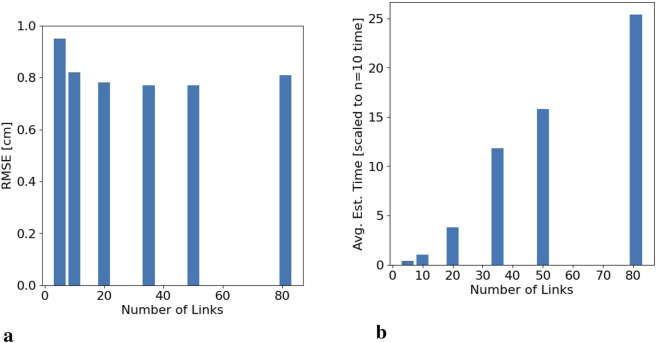
Effects of varying n across all configurations.**(a)** Root Mean Square Error for varying n . **(b)** Estimation time required for different number of links, scaled by the time required for n=10 **(a)** RMSE for varying number of links. **(b)** Average estimate time for varying number of links. The y-axis is scaled w.r.t the computation time of n=10 value to illustrate relative changes.

Figure 4 illustrates the impact of a changing n on the RMSE of the model and the average time required by the model to estimate the configuration. It is clear that while there is accuracy improvement as n increases, it is less than an order of magnitude and provides diminishing returns with link segments smaller than 4 cm, illustrated in Figure 4a. However, the computation time required to estimate the parameters increases rapidly as the number of links increases, shown in Figure 4b. As a result, for the cases in this experiment, utilizing a level of cable discretization above n=20 is not worth the computational cost. For cases evaluating performance in this article, n was set to 10 or 20.

### 3.4 Prediction performance

Previous sections demonstrate the increased model performance when incorporating parameter estimation. However, these sections do not explore positions after α has been calculated. To be a viable planning tool for in-space robotics, the model must be capable of predicting behavior for desired positions that are not accompanied by concurrent measurements.

**FIGURE 5:**
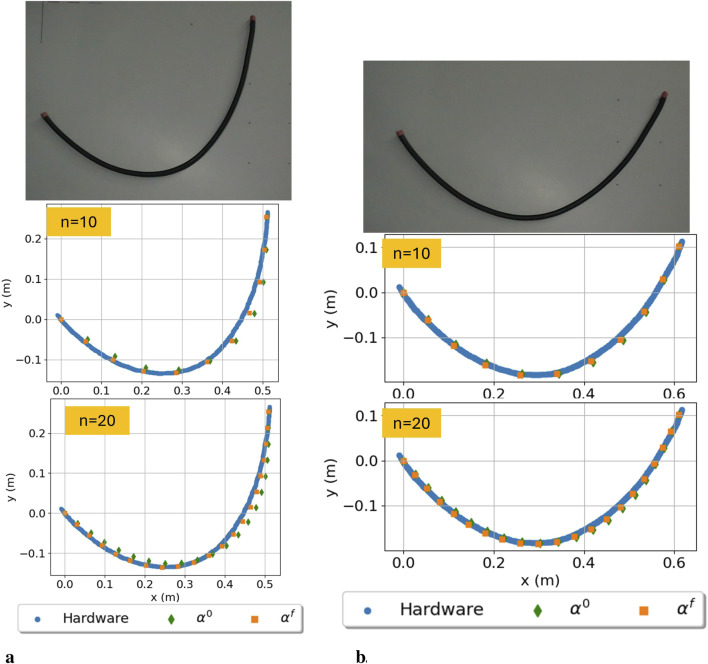
Hardware experiment data and corresponding prediction estimate results from Equation 8 compared to hardware data for n=10,20 . The green diamonds are the estimates based on the initial parameters $\alpha^{0}$ , while the orange squares are the solution including the calculated $\alpha^{f}$ . **(a)** Desired end position of (0.51,0.25)[m] . **(b)** Desired end position of (0.61,0.10)[m] **(a)** $p_{\mathrm{des}}=\left(0.51,0.25\right)\left[m\right]$ **(b)** $p_{\mathrm{des}}=\left(0.61,0.10\right)\left[m\right]$ .

Two configurations were chosen as a test set to validate the calculation of parameters for the cable when it is moved to a variety of positions. The parameter estimation was computed again using only 10 configurations, m=10 , to give the estimation of α no knowledge of the test set configurations. Figure 5 illustrates the results of the test cases evaluated, including the potential energy solution with $\alpha_{0}$ and with the calculated α including the non-zero reference configuration.

The results of the test set have increased accuracy by including the parameters, as shown in Table 6. For both cases, the RMSE from utilizing a pre-calculated α is halved from the initial solution with $\alpha^{0}$ . The RMSE is the same magnitude as when these sets are included in the calculation of α (as in Table 4); for n=10 , the RMSE of the configurations in Figures 5a,b are 5 mm and 3 mm, respectively.

**TABLE 6:** Root Mean Square Error (RMSE) for evaluated cable used for parameter estimation: using $\alpha^{0}$ , when included in the Least Squares (LS) calculation of α , and with loading in a previously estimated $\alpha^{f}$ .

End Pose [m]	n	RMSE [m]		
		$\alpha^{0}$	LS	$\alpha^{f}$
(0.51, 0.25)	10	0.012	0.005	0.005
(0.51, 0.25)	20	0.012	0.005	0.004
(0.61, 0.10)	10	0.006	0.003	0.003
(0.61, 0.10)	20	0.005	0.002	0.002

This work demonstrates promising results towards a multibody spring model of cables as DLOs that accounts for non-zero equilibrium configurations. For the cases evaluated here, the proposed model is able to predict positions across the length of the cable as the end position is moved - which is a valuable advancement for manipulation planning of cables.

## 4 Discussion

The results of this work illustrate the challenges with evaluating DLOs, particularly electrical cables, which often contain uneven cross sections and internal stresses contributing to a bent equilibrium position. Estimating the effective stiffness and equilibrium positions of a stiff DLO greatly inform robotics trajectory planning and control algorithms by improving the accuracy of the prediction model. The parameter estimation has a clear positive impact on the model performance for a static DLO configuration. The parameterization of the cable into a discrete number of links allows for the designer to have control over the size of the state space in the model. For the cable evaluated in this article, it was not significantly beneficial to have links smaller than 4 cm. This is likely a factor of the cable stiffness and diameter.

The DLO problem posed in this article focuses on one set of constraints and boundary conditions that might occur for in-space or lunar DLO operations: quasi-static, obstacle-free, planar translation with pinned ends. The addition of angle constraints, obstacles, environmental factors, and maximum bend constraints further complicate the potential energy problem. The quasi-static nature of this work may not be applicable to all DLO manipulation operations, a robotic trajectory plan may require better knowledge of DLO velocity kinematics or dynamics. The image processing procedure was only done for 2D images, making the estimation of out of plane bending difficult. As a result, while the formulation presented in this article utilized transformation matrices that allow for 3D kinematics, the generalized coordinates consisted of one rotation DOF per joint since there is no way to estimate out of plane values.

### 4.1 Future work

The model developed in this article informs future work to advance robotic cable manipulation. Dynamics are of significant interest, as well as the addition of parameters that impact cable energy, shape, and path planning. For DLO operations, additional parameters and constraints such as bend requirements, obstacle avoidance, and boundary conditions are required, in addition to varying external forces such as gravity or interactions with fixture points. For in-space systems, it is important to understand the constraint forces and how the manipulation of the DLO imparts forces back to the larger space systems. The derivation of such forces is more relevant to environments of increased complexity and dynamic models, and is therefore left to future work.

An additional area of future work is to increase the rotational DOF at each joint, enabling out of plane bending. Coupled with improved data processing to measure the 3D centerline, the increased DOF allows for the parameter estimation to account for out of plane configurations, not uncommon in electrical cables after storage. However, adding additional joint DOF increases the number of states by n for each added DOF, and will require an additional study into trade-off between accuracy and size of the state space. Force sensor data on an end effector would supplement the visual segmentation data, but would conversely increase the computational requirements in the system.

An interesting avenue of further investigation is the definition of the loss function for estimating α . The standard least squares residual utilized in this article demonstrates the feasibility of the parameter estimation scheme with batch estimation, but a comparison with sequential estimation and probability based approaches may increases performance and be better suited for more complex DLO manipulation tasks.

### 4.2 Conclusion

Manipulation of DLOs is a crucial technology to advancing robotic construction and maintenance across all industries. For in-space applications there are unique challenges that limit computational ability, increase risk, and reduce the viability a fully teleoperated system. In order to have a robotic construction and maintenance fleet for in-space applications, it is imperative the space domain develop an architecture that embraces the challenge of DLOs. In an extreme environment such as space, a DLO must be tethered at both ends, making it challenging to observe an unconstrained equilibrium position.

This article presents an evaluation of the trade-off between model accuracy and required state space size. This trade-off is not explored in the literature, and is important to in-space operations where there are limitations on available computational resources. The parametric multibody model allows for complexity to become a design parameter and it is shown that for the obstacle-free configurations evaluated in this work, increasing the number of states does not proportionally decrease distance error; increasing the number of states from 10 to 20 reduces RMSE by 0.4 mm, but takes 4 times as long to compute. The methodology for parameter estimation presented in this article is a novel approach to estimating the stiffness and equilibrium position of DLOs, increasing model accuracy by an average of 28% without increasing the size of the state space. The model-based formulation and parameter estimation presented in this article move the domain of in-space construction closer to a reality, and contribute to the rapid advancement of large-scale space missions.

## Data Availability

The raw data supporting the conclusions of this article will be made available by the authors, upon request.

## References

[B1] Belvin W. K. Doggett W. R. Watson J. J. Dorsey J. T. Warren J. E. Jones T. C. (2016). “enIn-space structural assembly: applications and technology,” in 3rd AIAA spacecraft structures conference (San Diego, California, USA: American Institute of Aeronautics and Astronautics). 10.2514/6.2016-2163

[B2] Bradski G. (2000). The OpenCV library. Dr. Dobb’s Journal of Software Tools. Available online at: https://github.com/opencv/opencv/wiki/CiteOpenCV.

[B3] Branch M. A. Coleman T. F. Li Y. (1999). A subspace, interior, and conjugate gradient method for large-scale bound-constrained minimization problems. SIAM J. Sci. Comput. 21, 1–23. 10.1137/s1064827595289108

[B4] Caporali A. Galassi K. Zanella R. Palli G. (2022). FASTDLO: fast deformable linear objects instance segmentation. IEEE Robotics Automation Lett. 7, 9075–9082. 10.1109/lra.2022.3189791

[B5] Caporali A. Kicki P. Galassi K. Zanella R. Walas K. Palli G. (2024). Deformable linear objects manipulation with online model parameters estimation. IEEE Robotics Automation Lett. 9, 2598–2605. 10.1109/lra.2024.3357310

[B6] Chang M. L. Marquez J. J. (2018). Human-automation allocations for current robotic space operations: space station remote manipulator system.

[B7] Choe B. Choi M. G. Ko H.-S. (2005). “Simulating complex hair with robust collision handling,” in *Proceedings of the 2005 ACM SIGGRAPH/Eurographics symposium on Computer animation* (ACM), 153–160. 10.1145/1073368.1073389

[B8] Cresta C. Rajaram R. McQuarry A. K. Avila T. V. Vaughan M. P. Cooper J. R. (2024). “Autonomous robotic manipulator software,” in Aiaa aviation forum and ascend 2024 (American Institute of Aeronautics and Astronautics). 10.2514/6.2024-4911

[B9] Deul C. Kugelstadt T. Weiler M. Bender J. (2018). Direct position-based solver for stiff rods. Comput. Graph. Forum 37, 313–324. 10.1111/cgf.13326

[B10] Everson H. Moser J. Quartaro A. Glassner S. Komendera E. (2020). Autonomous multi-robot assembly of solar array modules: experimental analysis and insights in 2020 IEEE/RSJ International Conference on Intelligent Robots and Systems (IROS) (IEEE). 10.1109/iros45743.2020.9341298

[B11] Gao Y. Chien S. (2017). Review on space robotics: toward top-level science through space exploration. Sci. Robotics 2, eaan5074. 10.1126/scirobotics.aan5074 33157901

[B12] Gazzola M. Dudte L. H. McCormick A. G. Mahadevan L. (2018). Forward and inverse problems in the mechanics of soft filaments. R. Soc. Open Sci. 5, 171628. 10.1098/rsos.171628 30110439 PMC6030325

[B13] Govindaraj S. Gancet J. Urbina D. Brinkmann W. Aouf N. Lacroix S. (2019). “Pro-act: planetary robots deployed for assembly and construction of future lunar isru and supporting infrastructures,” in Advanced space technologies in robotics and automation (ASTRA) 2019.

[B14] Hammond M. Dempsey A. Ward W. Stewart S. Neilan J. H. Friz J. (2023). A hybrid soft material robotic end-effector for reversible in-space assembly of strut components. Front. Robotics AI 10, 1099297. 10.3389/frobt.2023.1099297 PMC10354789 37476205

[B15] Hermansson T. Bohlin R. Carlson J. S. Söderberg R. (2013). Automatic assembly path planning for wiring harness installations. J. Manuf. Syst. 32, 417–422. 10.1016/j.jmsy.2013.04.006

[B16] Hu J. Jones D. Dogar M. R. Valdastri P. (2024). Occlusion-robust autonomous robotic manipulation of human soft tissues with 3-d surface feedback. IEEE Trans. Robotics 40, 624–638. 10.1109/tro.2023.3335693

[B17] Iben H. Meyer M. Petrovic L. Soares O. Anderson J. Witkin A. (2013). “Artistic simulation of curly hair,” in *Proceedings of the 12th ACM SIGGRAPH/eurographics Symposium on computer animation* (ACM). 10.1145/2485895.2485913

[B18] ICON (2022). Icon to develop lunar surface construction system with $57.2 million nasa award

[B19] Jiang X. mo Koo K. Kikuchi K. Konno A. Uchiyama M. (2010). “Robotized assembly of a wire harness in car production line,” in 2010 IEEE/RSJ International Conference on Intelligent Robots and Systems (IEEE). 10.1109/iros.2010.5653133

[B20] Jin S. Lian W. Wang C. Tomizuka M. Schaal S. (2022). Robotic cable routing with spatial representation. IEEE Robotics Automation Lett. 7, 5687–5694. 10.1109/lra.2022.3158377

[B21] Karumanchi S. Edelberg K. Nash J. Bergh C. Smith R. Emanuel B. (2018). Payload‐centric autonomy for in‐space robotic assembly of modular space structures. J. Field Robotics 35, 1005–1021. 10.1002/rob.21792

[B22] Koessler A. Filella N. R. Bouzgarrou B. Lequievre L. Ramon J.-A. C. (2021). “An efficient approach to closed-loop shape control of deformable objects using finite element models,” in 2021 IEEE International Conference on Robotics and Automation (ICRA) (IEEE), 1637–1643. 10.1109/icra48506.2021.9560919

[B23] Lan P. Shabana A. A. (2009). Integration of b-spline geometry and ANCF finite element analysis. Nonlinear Dyn. 61, 193–206. 10.1007/s11071-009-9641-6

[B24] Leibrandt K. da Cruz L. Bergeles C. (2023). Designing robots for reachability and dexterity: continuum surgical robots as a pretext application. IEEE Trans. Robotics 39, 2989–3007. 10.1109/tro.2023.3275381

[B25] Li W.-J. Cheng D.-Y. Liu X.-G. Wang Y.-B. Shi W.-H. Tang Z.-X. (2019). On-orbit service (OOS) of spacecraft: a review of engineering developments. Prog. Aerosp. Sci. 108, 32–120. 10.1016/j.paerosci.2019.01.004

[B26] Liu F. Su E. Lu J. Li M. Yip M. C. (2023). Robotic manipulation of deformable rope-like objects using differentiable compliant position-based dynamics. IEEE Robotics Automation Lett. 8, 3964–3971. 10.1109/lra.2023.3264766

[B27] Lv N. Liu J. Ding X. Liu J. Lin H. Ma J. (2017). Physically based real-time interactive assembly simulation of cable harness. J. Manuf. Syst. 43, 385–399. 10.1016/j.jmsy.2017.02.001

[B28] Lv N. Liu J. Jia Y. (2022). Dynamic modeling and control of deformable linear objects for single-arm and dual-arm robot manipulations. IEEE Trans. Robotics 38, 2341–2353. 10.1109/tro.2021.3139838

[B29] Lv N. Liu J. Xia H. Ma J. Yang X. (2020). A review of techniques for modeling flexible cables. Computer-Aided Des. 122, 102826. 10.1016/j.cad.2020.102826

[B30] Merila J. R. Neubert J. Mahlin M. (2023). “Scaling climbing collaborative mobile manipulators for outfitting a tall lunar tower and truss structures,” in Ascend 2023 (American Institute of Aeronautics and Astronautics). 10.2514/6.2023-4755

[B31] Monguzzi A. Dotti T. Fattorelli L. Zanchettin A. M. Rocco P. (2025). Optimal model-based path planning for the robotic manipulation of deformable linear objects. Robotics Computer-Integrated Manuf. 92, 102891. 10.1016/j.rcim.2024.102891

[B32] Muthumanickam N. K. Pless S. Rothgeb S. Davis J. (2022). Industrialized and robotic construction advances in terrestrial construction and opportunities in space construction

[B33] Nakanose S. Nakamura-Messenger K. (2023). “Gitai USA: providing safe and affordable means of labor in space,” in Ascend 2023 (American Institute of Aeronautics and Astronautics). 10.2514/6.2023-4744

[B34] NASA (2024). Architecture definition document – revision b. Available online at: https://www.nasa.gov/moontomarsarchitecture-architecturedefinitiondocuments/.

[B35] Olson G. Hatton R. L. Adams J. A. Mengüç Y. (2020). An euler–Bernoulli beam model for soft robot arms bent through self-stress and external loads. Int. J. Solids Struct. 207, 113–131. 10.1016/j.ijsolstr.2020.09.015

[B36] Puig-Navarro J. Bisio D. R. Pye J. E. Granov Y. Moser J. N. Friz J. S. (2024). “A trajectory tracking algorithm for the lsms family of cable-driven cranes,” in Robotics: science and systems (RSS) (Delft, Netherlands), 1–12.

[B37] Quartaro A. Cooper J. R. Komendera E. E. (2023). “Robotic software architecture for in-space outfitting operations,” in Ascend 2023 (American Institute of Aeronautics and Astronautics). 10.2514/6.2023-4681

[B38] Quartaro A. Cooper J. R. Moser J. N. Komendera E. E. (2024). “Modeling deformable linear objects for autonomous robotic outfitting of lunar surface systems,” in ASCE Earth and space 2024 (American Institute of Aeronautics and Astronautics).

[B39] Rojas D. (2022). Autonomous robotic manipulation of deformable linear objects during deep space maintenance and repair procedures. Master’s thesis. Available online at: https://escholarship.org/uc/item/0dz2z0df.

[B40] Shah A. Blumberg L. Shah J. (2018). Planning for manipulation of interlinked deformable linear objects with applications to aircraft assembly. IEEE Trans. Automation Sci. Eng. 15, 1823–1838. 10.1109/tase.2018.2811626

[B41] Suzuki R. Okada Y. Yokota Y. Saijo T. Eto H. Sakai Y. (2022). Cooperative towing by multi-robot system that maintains welding cable in optimized shape. IEEE Robotics Automation Lett. 7, 11783–11790. 10.1109/lra.2022.3183529

[B42] Tang T. Fan Y. Lin H.-C. Tomizuka M. (2017). “State estimation for deformable objects by point registration and dynamic simulation,” in 2017 IEEE/RSJ International Conference on Intelligent Robots and Systems (IROS) (IEEE). 10.1109/iros.2017.8206058

[B43] Tummers M. Lebastard V. Boyer F. Troccaz J. Rosa B. Chikhaoui M. T. (2023). Cosserat rod modeling of continuum robots from Newtonian and Lagrangian perspectives. IEEE Trans. Robotics 39, 2360–2378. 10.1109/tro.2023.3238171

[B44] Virtanen P. Gommers R. Oliphant T. E. Haberland M. Reddy T. Cournapeau D. (2020). SciPy 1.0: fundamental algorithms for scientific computing in Python. Nat. Methods 17, 261–272. 10.1038/s41592-019-0686-2 32015543 PMC7056644

[B45] Wakamatsu H. Hirai S. Iwata K. (1995). “Modeling of linear objects considering bend, twist, and extensional deformations,” in Proceedings of 1995 IEEE International Conference on Robotics and Automation (IEEE), 1 433–438. 10.1109/robot.1995.525322

[B46] Waltz W. J. Grande M. L. Moses R. W. (2022). “Autonomous system operations for lunar safe haven establishment and sustainment,” in AIAA SCITECH 2022 forum (American Institute of Aeronautics and Astronautics). 10.2514/6.2022-2075

[B47] Xun L. Zheng G. Kruszewski A. (2024). Cosserat-rod-based dynamic modeling of soft slender robot interacting with environment. IEEE Trans. Robotics 40, 2811–2830. 10.1109/tro.2024.3386393

[B48] Yan M. Zhu Y. Jin N. Bohg J. (2020). Self-supervised learning of state estimation for manipulating deformable linear objects. IEEE Robotics Automation Lett. 5, 2372–2379. 10.1109/lra.2020.2969931

[B49] Ying C. Yamazaki K. (2024). Obstacle avoidance shape control of deformable linear objects with online parameters adaptation based on differentiable simulation. ROBOMECH J. 11, 15. 10.1186/s40648-024-00283-1

[B50] Yu M. Lv K. Wang C. Tomizuka M. Li X. (2023). “A coarse-to-fine framework for dual-arm manipulation of deformable linear objects with whole-body obstacle avoidance,” in 2023 IEEE International Conference on Robotics and Automation (ICRA) (IEEE), 10153–10159. 10.1109/icra48891.2023.10160264

[B51] Zhang L. Schuler J. Dokos A. Xu Y. Bell E. Muller T. (2024). “Isru pilot excavator wheel testing in lunar regolith simulant,” in Earth and space 2024 (American Society of Civil Engineers), 173–187. 10.1061/9780784485736.016

[B52] Zhang T. Y. Suen C. Y. (1984). A fast parallel algorithm for thinning digital patterns. Commun. ACM 27, 236–239. 10.1145/357994.358023

[B53] Zhaole S. Zhu J. Fisher R. B. (2024). “Dexdlo: learning goal-conditioned dexterous policy for dynamic manipulation of deformable linear objects,” in 2024 IEEE International Conference on Robotics and Automation (ICRA) (IEEE), 35 16009–16015. 10.1109/icra57147.2024.10610754

